# Does exogenous female sex hormone administration affect the rate of tooth movement and root resorption? A systematic review of animal studies

**DOI:** 10.1371/journal.pone.0257778

**Published:** 2021-09-28

**Authors:** Eleftherios G. Kaklamanos, Miltiadis A. Makrygiannakis, Athanasios E. Athanasiou

**Affiliations:** 1 Hamdan Bin Mohammed College of Dental Medicine, Mohammed Bin Rashid University of Medicine and Health Sciences, Dubai, United Arab Emirates; 2 Department of Orthodontics, School of Dentistry, National and Kapodistrian University of Athens, Athens, Greece; 3 Department of Dentistry, European University Cyprus, Nicosia, Cyprus; University of Zurich, SWITZERLAND

## Abstract

**Background:**

The long-term use of contraceptive methods that contain estrogens, progestogens or combinations of the above among women aged 15 to 49 years is extensive. Both estrogens and progestogens affect bone metabolism.

**Objective:**

To systematically investigate and appraise the quality of the available evidence from animal studies regarding the impact of exogenous administration of female sex hormones on the rate of orthodontic tooth movement and root resorption.

**Search methods:**

Search without restriction in seven databases (including grey literature) and hand searching were performed until May 2021.

**Selection criteria:**

We looked for controlled animal studies investigating the effect from exogenous administration of formulations containing female sex hormones on the rate of orthodontic tooth movement and root resorption.

**Data collection and analysis:**

After study retrieval and selection, relevant data was extracted, and the risk of bias was assessed using the SYRCLE’s Risk of Bias Tool. The quality of available evidence was assessed with the Grades of Recommendation, Assessment, Development, and Evaluation.

**Results:**

Three studies were identified, all being at unclear risk of bias. Overall, administration of progesterone and the combinations of estradiol with norgestrel and desogestrel were shown to significantly decrease the rate of orthodontic tooth movement when given for longer periods (>3 weeks). Inconsistent information was detected for shorter periods of consumption. Estradiol, with desogestrel use, resulted in less root resorption. The quality of the available evidence was considered to be low.

**Conclusions:**

Exogenous administration of female sex hormones may decelerate in the long term the rate of tooth movement and decrease orthodontically induced root resorption in animals. Until more information becomes available, an orthodontist should be able to identify a patient consuming such substances and understand the potential clinical implications and adverse effects that may arise.

**Registration:**

PROSPERO: CRD42017078208; https://clinicaltrials.gov/.

## Introduction

In many parts of the world, a significant percentage of women have, during their lifetime, used contraception for long periods of time [1,2]. In the United States, more than 25% of women aged 15 to 49 years old are currently using oral contraceptive pills, long-acting reversible contraceptives, or other methods of contraception that contain various estrogens, progestogens or combinations [3]. Both estrogens and progestogens have been shown to affect bone turnover [4].

Exogenous estrogens and progestogens are not only used for hormonal birth control, but also as menopausal hormone therapy. Treatment with estrogens alone in hysterectomized women or in combination with a progestogen in non-hysterectomized women is indicated for the prevention of postmenopausal osteoporosis, as well as the treatment of menopausal symptoms [5]. Estrogens’ administration is indicated in cases of vasomotor symptoms [6] and related genitourinary symptoms of menopause [7]. Estrogens and progestogens can also be used in the treatment of gynecological conditions, like endometrial hyperplasia [8], uterine bleeding [9] and premenstrual syndrome [10], to address infertility [11], as well as to support pregnancy by preventing miscarriage and preterm delivery [12,13].

In the field of orthodontics, thorough documentation of medical history, along with clinical examination and diagnostic records, are always imperative [14]. Specific information on any medication administered is not only significant in recording information about a patient’s health status but might also provide further insights on clinical treatment as well. Medication administration may have varying effects on the rate of orthodontic tooth movement, during active treatment or the post-treatment period, as well as the associated root resorption development [15–20]. Thus, clinicians must be capable of identifying their patients’ medication consumption patterns and understand the potential implications for their treatment.

Information relevant to the impact of exogenous administration of female sex hormones on the rate of orthodontic tooth movement and root resorption should ideally be retrieved from clinical trials. However, due to ethical reasons and difficulties in recruiting an adequate sample, there is a lack of human studies on this topic. As an alternative, animal experiments are expected to include pertinent information, while circumventing the ethical and practical considerations associated with experimentations on human subjects [21]. Murine reproductive research models have been used extensively for this reason [22]. This is because the estrus cycle of rats is analogous to the human menstrual cycle, not only in terms of the maintenance mechanism, but also regarding hormonal control [23].

### Objective

The aim of this systematic review was to systematically investigate and assess the quality of the existing available scientific information regarding the rate of tooth movement and orthodontically induced root resorption following the systemic administration of exogenous female sex hormones.

## Materials and methods

### Protocol and registration

The present review was based on a protocol developed, registered, carried out and reported following relevant methodological guidelines (PROSPERO: CRD42017078208) (S1 Checklist) [24–27]. As the present study is a systematic review, ethical approval was not necessary.

### Eligibility criteria

The Participant, Intervention, Comparator, Outcomes and Study design domains were used to describe the eligibility criteria (Table 1). We looked for prospective experimental controlled studies on healthy female animal subjects, assessing the rate of tooth movement and/or root resorption following the administration of exogenous female sex hormones. All types of orthodontic interventions to induce movement of teeth were considered, and the studies had to report on the amount of tooth movement either during the application of orthodontic forces or after their cessation. Tooth movement could be measured in various ways (with calipers, feeler gauges, etc. directly or from plaster models; from histological cuts directly on the optical microscope or from digital photos; radiographs of any kind i.e., lateral cephalometric radiographs, Cone Beam CT, micro-CT, etc.). The amount and extent of root resorption could be investigated histomorphometrically, by scanning electron or 3D surface microscopy or radiographically. Studies on male animals, as well as female animals under medication, with dietary deficiencies, co-morbidities or ovariectomy were excluded. Studies involving animals with additional clinical interventions such as tooth extraction, etc. were also eliminated, as well as studies presenting qualitative assessments. In terms of study design, we planned to select only experimental controlled animal studies and to exclude human, in vitro, ex-vivo or in silico studies; non-comparative studies, reviews, systematic reviews, meta-analyses and studies with fewer than 5 subjects per group analyzed, as per relevant methodological guidelines regarding the consideration of degrees of freedom for treatment comparisons [28,29].

**Table 1:** Eligibility criteria.

Domain	Inclusion criteria	Exclusion criteria
**Participants**	• Animal subjects undergoing any kind of active orthodontic tooth movement or after the cessation of orthodontic forces.	• Interventions involving additional interventions, e.g., tooth extraction. • Subjects who present co-morbidities, e.g., osteoporosis.
**Interventions**	• Systemic administration of female hormones (i.e., per os, intravenously, intraperitoneally, etc.).	• Non-systemic administration. • Administration of substances that are experimental or not prescribed to humans. Simultaneous administration of substances not considered in the present review. • Studies where the active substance or another intervention induced a pathological condition, studies with comorbidities or other conditions.
**Comparisons**	• Placebo intervention or no intervention.	
**Outcomes**	• Quantitative data regarding the rate of tooth movement [i.e., the amount of tooth movement in a specific period of time] measured by various ways [callipers, feeler gauges, lateral cephalometric radiographs, Cone Beam CT, etc.]. • Quantitative data regarding the amount and extent of root resorption [number of lacunae, area of lacunae, percentage of resorptive sites compared to the total root area, depth of lacunae, volume of lacunae, etc.] measured by various ways [histomorphometrically, by scanning electron or 3D surface microscopy, radiographically, etc.].	• Qualitative assessments regarding the rate of tooth movement. • Qualitative assessments regarding the amount and extent of root resorption associated with orthodontic tooth movement. • Inadequate definition of outcomes.
**Study design**	• Experimental prospective controlled studies (according to the Scottish Intercollegiate Guidelines Network algorithm for classifying study design (available at http://www.sign.ac.uk/assets/study_design.pdf).	• Non-comparative studies. *In vitro* or *ex-vivo* studies. Reviews, systematic reviews and meta-analyses. • Less than 5 subjects per group analyzed.

### Information sources and search strategy

The total content, in 7 databases, (Medline [PubMed], CENTRAL [Cochrane Library; includes records from Embase, CINAHL, ClinicalTrials.gov, WHO’s ICTRP, KoreaMed, Cochrane Review Groups’ Specialized Registers, and records identified by handsearching], Cochrane Database of Systematic Reviews [Cochrane Library], Scopus, Web of Knowledge [including Web of Science Core Collection, KCI Korean Journal Database, Russian Science Citation Index, SciELO Citation Index and Zoological Record], Arab World Research Source [EBSCO] and ProQuest Dissertation and Theses [ProQuest]) was searched until May 22^nd^, 2021, with strategies developed by the first author based on the MEDLINE search (Table 2). No language, date, or status of publication restrictions were set, and efforts to obtain additional studies were made by reviewing the reference lists in reviews, included and excluded studies, as well as other related articles. Duplicates were removed using EndNote’s duplicate identification strategy (EndNote X9™, Clarivate™, Philadelphia, PA, USA) and then manually by EGK. The corresponding authors of potentially included records were to be contacted, if needed, in order to provide additional data.

**Table 2:** Strategy for database search [May 22^nd^, 2021].

Database	Search strategy	Hits
**PubMed** http://www.ncbi.nlm.nih.gov/pubmed	(orthodon*[tiab] OR “orthodontic force”[tiab] OR “mechanical force”[tiab]) AND ("tooth movement"[tiab] OR “orthodontic movement”[tiab] OR “orthodontic anchorage”[tiab] OR “root resorption”[tiab])	**4574**
**Cochrane Central Register of Controlled Trials** http://onlinelibrary.wiley.com/cochranelibrary	(orthodont* OR “orthodontic force” OR “mechanical force”) AND ("tooth movement" OR “orthodontic movement” OR “orthodontic anchorage” OR “root resorption”) in Record Title OR (orthodont* OR “orthodontic force” OR “mechanical force”) AND ("tooth movement" OR “orthodontic movement” OR “orthodontic anchorage” OR “root resorption”) in Abstract—(Word variations have been searched)	**2103**
**Cochrane Database of Systematic Reviews** http://onlinelibrary.wiley.com/cochranelibrary	(orthodont* OR “orthodontic force” OR “mechanical force”) AND ("tooth movement" OR “orthodontic movement” OR “orthodontic anchorage” OR “root resorption”) in Record Title OR (orthodont* OR “orthodontic force” OR “mechanical force”) AND ("tooth movement" OR “orthodontic movement” OR “orthodontic anchorage” OR “root resorption”) in Abstract—(Word variations have been searched)	**20**
**Scopus** https://www.scopus.com	TITLE-ABS ((orthodont* OR "orthodontic force" OR "mechanical force") AND ("tooth movement" OR "orthodontic movement" OR "orthodontic anchorage" OR "root resorption")) AND (LIMIT-TO (SUBJAREA,"DENT")) AND (LIMIT-TO (EXACTKEYWORD,"Tooth Movement"))	**1343**
**Web of Science™** http://apps.webofknowledge.com/	TITLE: ((orthodon* OR orthodontic force OR mechanical force) AND ("tooth movement" OR orthodontic movement OR orthodontic anchorage OR root resorption)) Timespan: All years. Search language = Auto	**1794**
**Arab World Research Source** http://0-web.a.ebscohost.com.amclb.iii.com	TI tooth movement OR AB tooth movement	**4**
**ProQuest Dissertations and Theses Global** http://search.proquest.com/dissertations	ti(orthodont* AND "tooth movement") AND ab(orthodont* AND "tooth movement") [Full text]	**141**

### Selection process

The titles and abstracts of the retrieved records, followed by the full report of any record considered to meet the inclusion criteria, were assessed independently and in duplicate by EGK and MAM, who were both not blinded. If the abstract was unclear, the full paper was accessed to determine eligibility for inclusion.

### Data collection and data items

Special data collection forms were used for data extraction, which was performed independently by the first two authors. The following information was recorded: bibliographic details; information on experimental design and verification of study eligibility; characteristics of the animals (number of animals in each group and sample size calculation; age and weight of animals) and tooth movement mechanics; data on medicinal administration; measurement of outcomes details (orthodontic tooth movement and/or root resorption) and reliability assessment; results. Results were to be extracted and categorized separately for each species or type of mechanics used, since differences are to be expected [30]. Results were also categorized depending on the time period of force application: less than 3 weeks, and 3 weeks or more. Orthodontic tooth movement has been shown to be divided into two phases, a non- linear phase (<3 weeks) and a linear phase thereafter, with the speed of movement peaking at 3 weeks following force application [30,31].

### Study risk of bias assessment

The risk of bias in individual studies was assessed with the SYRCLE’s risk of bias tool for animal studies [32] and the summary risk of bias within a study, according to Higgins and co-workers [26]. Assessments were subsequently entered into the Risk-of-bias VISualization (robvis) web application [33]. In all the aforementioned processes, disagreements were settled by discussion with AEA; following the relevant suggestions, kappa statistics were not calculated [26].

### Effect measures, synthesis methods, certainty assessment and additional analyses

Although a synthesis of the results was planned, it was ultimately not carried out due to methodological diversity [26]. Due to inadequate information, analyses for “small-study effects” and publication bias, as well as subgroup analyses, were not performed [26]. Finally, despite the lack of extensive information, the quality of available evidence regarding the effect of exogenous female hormone administration on the rate of tooth movement and root resorption after 3 weeks of force application was assessed with the Grades of Recommendation, Assessment, Development, and Evaluation in order to adopt a structured and transparent approach in formulating an interpretation of the evidence [34].

## Results

### Study selection

**Fig 1:**
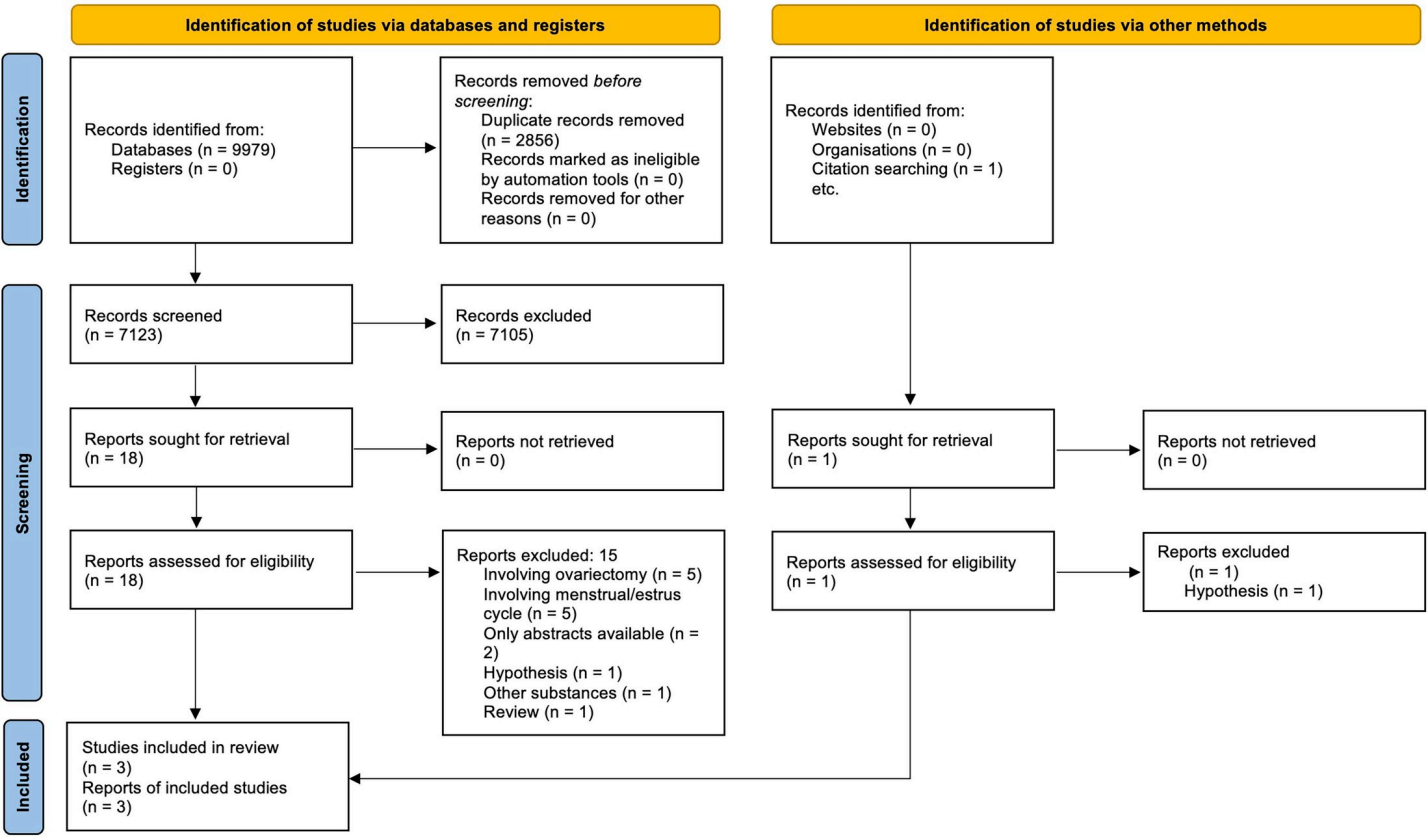
PRISMA 2020 flow diagram.

The flow of records through the reviewing process is shown in Fig 1. We initially identified 9980 references. We excluded 2856 as duplicates and another 7105 on the basis of their title and abstract. From the nineteen records that remained and were assessed for eligibility, sixteen records were excluded for the following reasons: papers investigating the effect of ovariectomy (5); papers investigating the phases of the menstrual/estrus cycle (5); studies where only the abstract was available (2); papers that constituted hypotheses only (2); a paper that was a review (1); a study that involved other substances (1) (Table 3). Finally, 3 study records were assessed to be eligible for inclusion [35–37].

**Table 3:** Excluded studies, with reasons.

Excluded studies	Reason
Davidovitch Z, Musich D, Doyle M. Hormonal effects on orthodontic tooth movement in cats—a pilot study. Am J Orthod. 1972;62:95–6.	Involving other substances
Deng L, Guo Y. Estrogen effects on orthodontic tooth movement and orthodontically-induced root resorption. Arch Oral Biol. 2020;118:104840.	Review
Guo J, Che XX, Zhao Q, Chen YX. [An experimental study on the relationship between the orthodontic tooth movements and menstrual cycle]. Shanghai Kou Qiang Yi Xue. 2007;16:187–91.	Involving menstrual cycle
Haruyama N, Igarashi K, Saeki S, Otsuka-Isoya M, Shinoda H, Mitani H. Estrous-cycle-dependent variation in orthodontic tooth movement. J Dent Res. 2002;81:406–10.	Involving estrus cycle
Haruyama N, Igarashi K, Saeki S, Otsuka-Isoya M, Shinoda H, Mitani H. Effects of continuous administration of 17b-estradiol on orthodontic tooth movement in rats. J Dent Res 2002;81:A217	Conference abstract
Jin Z, Ding Y, Li X. [Effects of estrogen on experimental tooth movement in osteoporosis rats]. Zhonghua Kou Qiang Yi Xue Za Zhi. 2000;35:55–7.	Involving ovariectomy
Ketani A, Hamamci O. Effects of estrogen deficiency on tooth movement after force application: an experimental study in ovariectomized rats. Acta Odontol Scand. 2007;65:319–23.	Involving ovariectomy
Li T, Zhou Z, Wang H, Lv C, Zhang C, Tao G, Li X, Zou S, Duan P. Effects of estrogen on root repair after orthodontically induced root resorption in ovariectomized rats. Am J Orthod Dentofacial Orthop. 2020 Aug;158(2):247-263.e1	Involving ovariectomy
Lingyong J, Chao W, Yuqiong W, Peng Z, Bing F. Does Gonadotropin Releasing Hormone Agonists plus add-back therapy bring an aurora to orthodontic treatment. Dent Hypotheses. 2012;3:95–98.	Hypothesis
Monghini EM, Park DM, Franci JAA, Barros VMR, Matsumoto MAN, Mizusaki CI. Influence of estrogen on dental movement due to orthodontic treatment. J Dent Res. 2001;80:1099.	Conference abstract
Seifi M, Ezzati B, Saedi S, Hedayati M. The Effect of Ovariectomy and Orchiectomy on Orthodontic Tooth Movement and Root Resorption in Wistar Rats. J Dent (Shiraz). 2015;16:302–9.	Involving ovariectomy
Tan Z, Zhao Q, Chen Y. The mutual effects between orthodontic tooth movement and estrous cycle or estrogen. Biol Rhythm Res. 2010;41:75–81.	Involving estrus cycle
Wang B, Yang X, Zhou JP, Feng G, Dai HW, Huang L. Orthodontic tooth movement at different stages of adolescent female menstrual cycle. Chinese J Tissue Eng Res 2014;15:2332–7	Involving menstrual cycle
Xu X, Zhao Q, Yang S, Fu G, Chen Y. A new approach to accelerate orthodontic tooth movement in women: Orthodontic force application after ovulation. Med Hypotheses. 2010;75:405–7.	Hypothesis
Yamashiro T, Takano-Yamamoto T. Influences of ovariectomy on experimental tooth movement in the rat. J Dent Res. 2001;80:1858–61.	Involving ovariectomy
Zhao Q, Tan Z, Guo J, Chen YX. [Influences of orthodontic tooth movement on estrous cycle and estrogen in rats]. Zhonghua Kou Qiang Yi Xue Za Zhi. 2006;41:90–1.	Involving menstrual cycle

### Study characteristics

The characteristics of the included studies are presented in Table 4. Two of the included studies experimented on rats [36,37], while the third study used rabbits [35]. The duration of force application varied from 2 to 60 days, and female sex hormones were administered for 9 weeks at maximum. In one study, the administration of hormones started one week before the initiation of tooth movement [36], and 6 weeks earlier in another [35]. Orthodontic movement was induced either by reciprocal stainless-steel springs on the incisors [35,36] or by closed coil NiTi spring between incisors and the first molar [37]. The forces exerted ranged between 30-50g.

**Table 4:** Characteristics of the studies included in the systematic review.

Active substance & study	Subjects & tooth movement model [species; gender; age; weight]	Group characteristics^§^ [no; substance; dosage; route; administration]	Outcomes and methodology	Results*	
				OTM <3w	OTM >3w
**Progesterone** Poosti et al. [2009] [35]	Albino rabbits; female; 2m; 1850g Reciprocal SS springs on CIs [50g] **Force application:** 3w (1 activation)	**EG**_**1**_: 6; progesterone [5000μg]; IM; every day for 3w **EG**_**2**_: 5; progesterone [5000μg]; IM; every day for 9w **CG**: 8 **Sample size calculation:** No **Medication administration:** 3w [short term]/9w [long term]	**OTM:** Caliper; distance between the mesial corners of Mx Is	**EG**_**1**_: 1, 2w: ND **EG**_**2**_: 1, 2w: ND	**EG**_**1**_: 3w: ND**EG**_**2**_: 3w: ↓ in EG
**Estradiol + Norgestrel** Olyaee et al. [2013] [36]	Wistar rats; female; 3m; 250 ±25g Reciprocal SS springs on CIs [30g] **Force application:** 2w (1 activation)	**EG:** 24; ethinyl estradiol [25μg] + norgestrel [250 μg]; PO; 5d/w **PG:** 24; saline; PO; daily **Sample size calculation:** No **Medication administration:** 1 before OTM + for 2w after	**OTM:** Caliper; distance between the mesial corners of Mx Is	2d, 1w: ND 2w: ↓ in EG	
**Estradiol + Desogestrel** Yu et al. [2019] [37]	Sprague-Dawley rats; female; 3m; 250 ±30g NiTi coil springs from Mx FM to CIs [50g] **Force application:** 4w	**EG:** 40; ethinyl estradiol [18μg] + desogestrel [90μg]; PO daily **PG:** 40; saline; PO; daily **Sample size calculation:** No **Medication administration:** 4w	**OTM:** Caliper; distance [central fossae of the Mx FM to SM]	1, 2w: ↓ in EG	3, 4w: ↓ in EG
			**RR:** Light microscopy [Η&Ε]; resorption lacunae area in mesial root	1, 2w: ↓ in EG	3, 4w: ↓ in EG

^§^Dosages were standardized to μg.

*All results shown represent comparisons with placebo (preferably) or no pharmacological intervention control groups after specific periods of orthodontic tooth movement.

CI: Central incisor; CG: Control group receiving no kind of pharmacological intervention, neither the vehicle plus the active substance nor the vehicle only; d: Days; EG: Experimental group that received pharmacological intervention, different than placebo; FM: First molar(s); Η&Ε: Hematoxylin and eosin; I: Incisor(s); IM: Intramuscular injection; Mx: Maxillary; m: Month; nd: No difference; OTM: Orthodontic tooth movement; PG: Placebo group receiving the vehicle preparation without the active substance; PO: Per os; RR: Root resorption; SM: Second molar(s); SS: Stainless steel.

The retrieved papers investigated the administration of progesterone [35] and the combinations of estradiol with the progestogens norgestrel [36] and desogestrel [37]. All studies reported the rate of tooth movement during the application of orthodontic forces, and only one assessed orthodontically induced root resorption [37]. The rate of tooth movement was measured clinically with calipers, and root resorption was assessed from histological cuts with light microscopy. No sample size calculations were performed, and only Yu et al. assessed the error of the method [37]. No study assessed tooth movement after the cessation of orthodontic forces.

## Risk of bias within studies

**Fig 2:**
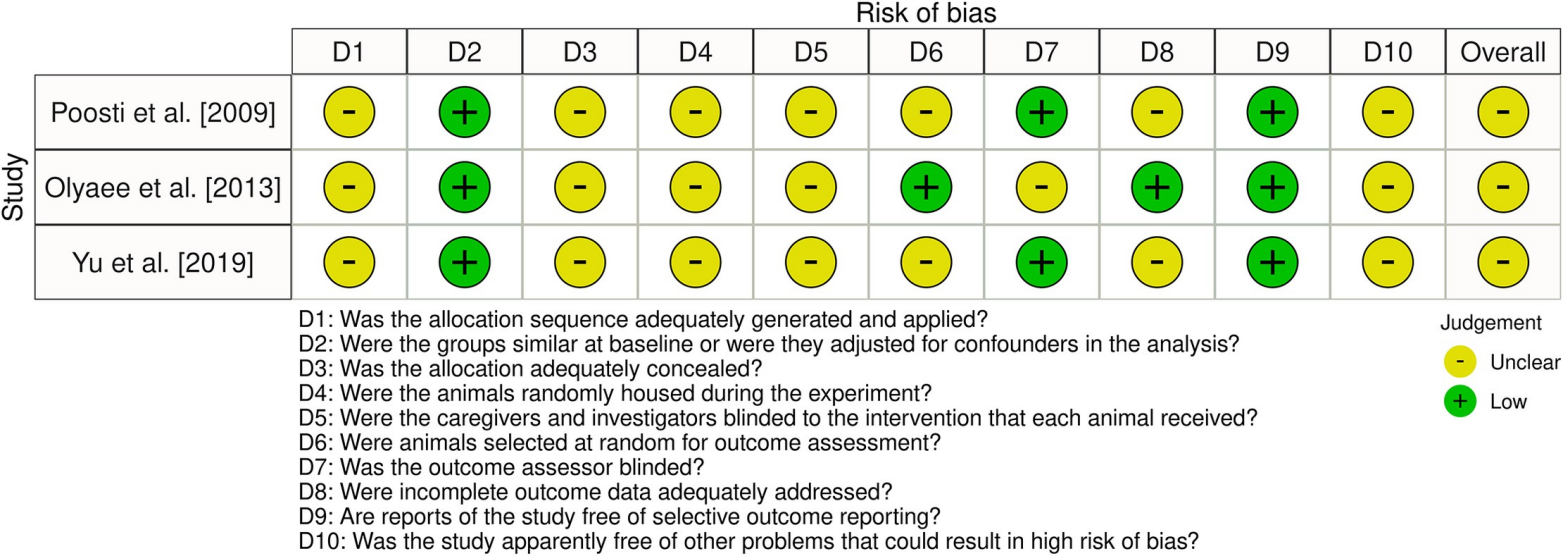
Risk of bias assessment.

All three studies were assessed to show an unclear risk of bias (Fig 2). All studies were considered to present low risk of bias regarding the items of group similarity and selective reporting, as well as unclear risk for random sequence generation, allocation concealment, random housing of the animals and blinding of caregivers and investigators. Random selection of animals for outcome assessment was assessed at low risk only for Olyaee et al. [36]. Assessor blinding was appraised to be at low risk for Poosti et al. [35] and Yu et al. [37], while the handling of incomplete data was at low risk only for Olyaee et al. [36].

### Effects of exogenous administration of female sex hormones

For the first two weeks after the application of orthodontic forces, progesterone administration in rabbits did not result in any statistically significant difference between the experimental and control group, even for the animals that started the treatment 6 weeks before the placement of the orthodontic appliance. In studies involving rats, the combination of estradiol with desogestrel produced a reduction in tooth movement after 2 weeks, whereas the combination with norgestrel resulted in significant differences in the measurements conducted in both the first and second weeks (Table 4).

Regarding the measurements after the third week of force application, Poosti et al. [35] demonstrated that progesterone resulted in significant decreases in the amount of tooth movement in rabbits, but only in the group that had been exposed to the exogenous hormone for 6 weeks before the initiation of movement. The combination of estradiol with desogestrel also led to a reduction in tooth movement measured in the rats 3 and 4 weeks after the commencement of the experiment [37] (Table 4).

The only study that included assessments of root resorption experimented on rats, and observed reductions in all measurements from the first until the fourth week of force application (Table 4).

Regarding the effect of exogenous female hormone administration on the rate of orthodontic tooth movement and orthodontically induced root resorption, the quality of available evidence was considered as low (Table 5).

**Table 5:** Quality of available evidence.

Quality assessment						Effect	Quality
Studies	Risk of bias	Inconsistency	Indirectness	Imprecision	Other		
**Progesterone [rate of tooth movement at 3 weeks]**							
1	Serious^1^	Not serious	Not serious^2^	Serious^3^	None	Decrease in the EG	⨁⨁◯◯ **LOW**
**Estradiol + Desogestrel [rate of tooth movement at 3 weeks]**							
1	Serious^1^	Not serious	Not serious^2^	Serious^3^	None	Decrease in the EG	⨁⨁◯◯ **LOW**
**Estradiol + Desogestrel [root resorption at 3 weeks]**							
1	Serious^1^	Not serious	Not serious^2^	Serious^3^	None	Decrease in the EG	⨁⨁◯◯ **LOW**

EG: Experimental group.

^1^Results were based on studies at unclear risk of bias

^2^Although results cannot be directly extrapolated to human clinical settings they are relevant to the animal setting

^3^The number of animals analysed was limited.

## Discussion

The long-term consumption of contraceptives that contain estrogens, progestogens or combinations of them among women aged 15 to 49 years is extensive [1–3]. Both estrogens and progestogens affect bone metabolism [4]. Overall, the hormonal formulations investigated resulted in reductions in the amount of tooth movement when administered for longer periods (>3 weeks). The same was noted for orthodontically induced root resorption. Although the quality of evidence assessment provides insight on the strength of the relevant recommendations, and the information provided is not fully translatable to human clinical scenarios, until more research information becomes available, it could be good practice to identify orthodontic patients’ consumption of similar medications and to keep in mind the potential clinical ramifications of such.

Oral contraceptive pills and long-acting reversible contraceptives include two main categories—progestin-only pills, and combinations of estrogens and progestogens [38]. Progestin-only pills function primarily through thickening the cervical mucus, and, secondarily, through inhibiting ovulation to variable degrees, reducing cilia activity in the fallopian tubes, and altering the endometrium [39]. Contraceptives containing both estrogens and progestogens function primarily though inhibition of ovulation via feedback mechanisms in the hypothalamic-pituitary-ovarian axis and thickening of the cervical mucus [38]. The papers used in the present review investigated the effect of progesterone [35] as well as combinations of estrogen with progestogens [36,37], and their results suggested reductions in the speed of orthodontically induced movement and root resorption when administered for longer periods.

Estrogen serum levels are of ovarian origin, and not only regulate female reproductive and sexual function [40], but are also indispensable in maintaining adequate bone mass and mineralization [41,42] through receptors detected in human cells [43]. Especially during pregnancy, estrogens have been found to exert a significant role regarding bone mass preservation [42]. Prevention of bone remodeling by estrogens is a result of osteoclastogenesis prevention from marrow precursors, as well as of induction of the Fas/FasL system that enhances osteoclast apoptosis [44,45]. Estrogens lead to a further inhibition in bone resorption through effects on the receptor activator of nuclear factor-Kappa B (RANK)/RANK ligand (RANKL)/osteoprotegerin (OPG) system and the production of some pro-resorptive cytokines (e.g., IL-1, IL-6, IL-7, TNF) [46–52]. Moreover, estrogens directly affect osteoblastic cells contributing to bone preservation [53,54].

Progesterone is a steroid sex hormone produced in the ovaries and involved in the menstrual cycle, pregnancy and embryogenesis. It has been shown to possess bone protective qualities [55], directly regulated by progesterone receptors in osteoblasts [56]. These are found in both osteoblasts and osteoclasts [57–59] and are upregulated by estrogen levels, possibly alluding to the fact that the bone effects of estrogens are being partially regulated via progestogens [43,57,58]. In addition, progesterone may participate in the control of bone matrix, through its inhibitory action on metalloproteinases [60,61]. Serum osteocalcin is significantly correlated with progesterone levels, indicating a further interdependence with bone-forming activities [62].

The combinations of estrogens with progestogens for hormonal contraception are extensively researched medications, as they were originally designed for long-term use by young, healthy women, unlike other drugs that are intended for therapeutic effect [63]. Regarding bone mineral metabolism, the effects of these formulations seem to differ between adolescent and adult women. In younger individuals, although this claim is contested, a small overall negative effect seems probable if their use commences very early after menarche. On the contrary, combined oral contraceptives seem to exert a positive effect on bone turnover in adult females [63]. Overall, there seems to be no association between the risk of fractures in the period before menopause and oral contraceptives use [64].

Results from studies on ovariectomized animals provide data that corroborate further the information located in the included studies. After surgical removal of the ovaries, there is a lack of endogenous progesterone and estrogens, resulting in bone loss that initiates in two weeks post-surgery at the earliest, reaches 50% approximately after 4 to 8 weeks, and becomes stable within 12 weeks [65]. Ovariectomy results in osteopenia and osteoporosis and, subsequently, a higher rate of tooth movement [66], while estrogen administration reduces the speed of movement in osteoporotic rats [67,68], as it is closely related to the activity of osteoclasts [69]. The absence of estrogens has been associated with greater root resorption following orthodontic tooth movement as well [68].

Although the information located from these animal studies was not extensive, some points might be relevant to the treatment of adolescent and adult females using contraceptive methods that involve exogenous hormone administration. An eventual protective effect against root resorption could be important. Given the potentially decelerating effect on tooth movement, an orthodontist should always consider the impact on space closure, frequency of the appointments, and estimation of the duration of orthodontic treatment. Both active and anchor segments’ resistance to movement might be increased, making space closure more challenging, and requiring altered biomechanical systems because of the altered bone turnover [70]. Furthermore, in some stages of treatment, there could be no issue in seeing patients at longer time intervals. Finally, it could be possible that the overall treatment duration might be increased in such circumstances. Although no direct information came out of the material studied, it could be possible that the retention period could be more uneventful in individuals receiving female sex hormones exogenously. Following the removal of orthodontic appliances, the mechanical equilibrium changes immediately, and leads to events analogous to those seen during active treatment, acting in opposite directions [71]. Possible implications could be magnified given the frequently long periods of consumption of such formulations in real life scenarios.

### Strengths and limitations

The employed search strategy was comprehensive, all-inclusive, up to May 2021 and followed established guidelines to reduce bias. Most importantly, as similar investigations might encounter significant practical obstacles in human subjects, the current review summarized the available information from animal models that have been used extensively in female reproduction research [22].

There also some limitations that ought to be further elaborated. The level of available evidence was assessed to be low, because of the peculiarities of the eligible studies, regarding nature, characteristics, and the retrieved data. In addition, we were not able to proceed with meta-analysis or other analyses due to the scarcity of information.

Furthermore, it should be highlighted that the presented information is applicable to animals and can be extrapolated to the clinic environment only indirectly. The administered dosages and durations of the tested formulations differ from those used in humans [72]. Also, the route of administration differs, which might lead to different pharmacokinetics and bioavailability [73]. Since different animal species and strains were used in the reviewed experiments, it is important to expect variability in the content and the properties of the bony tissue [74]. The specific biomechanics used to produece the movement of the teeth in the animal experiments are not comparable directly to those used in humans. In addition, in the clinic a patient’s medical status may affect the homeostasis of the periodontal ligament and the movement of the teeth [75,76]. Finally, the eligible studies did not include power sample calculations, possibly leading to problems regarding the precision of the presented results. Thus, the extent of the effect from exogenous female sex hormones administration on orthodontic tooth movement and root resorption is still unclear in human beings. However, executing such studies in humans could prove to be challenging, both ethically and practically.

### Recommendations for future research

Since female sex hormones are used widely nowadays among female adolescents and adults, further research is warranted regarding their effect on the phenomena associated with tooth movement and orthodontically induced root resorption. Future studies should follow relevant guidelines to improve standardization and control the various sources of bias. Finally, to increase generalizability parameters like dosages, duration, and routes of administration, together with the mechanics of movement should be close, as possible, to daily clinical practice.

## Conclusions

Based on the compiled information from animal studies, the exogenous female sex hormone administration might result in reductions in orthodontically induced tooth movement and root resorption when administered for longer periods. Although results that derive from animal studies cannot be directly extrapolated to human clinical scenarios, the clinician needs to be aware of the relevant implications in orthodontic treatment.

## Supporting information

S1 Checklist PRISMA 2020 checklist. (DOCX)
